# Characteristics of Marijuana Use During Pregnancy — Eight States, Pregnancy Risk Assessment Monitoring System, 2017

**DOI:** 10.15585/mmwr.mm6932a2

**Published:** 2020-08-14

**Authors:** Jean Y. Ko, Kelsey C. Coy, Sarah C. Haight, Tamara M. Haegerich, Letitia Williams, Shanna Cox, Rashid Njai, Althea M. Grant

**Affiliations:** ^1^Division of Reproductive Health, National Center for Chronic Disease Prevention and Health Promotion, CDC; ^2^Oak Ridge Institute for Science and Education, Oak Ridge, Tennessee; ^3^Division of Overdose Prevention, National Center for Injury Prevention and Control, CDC; ^4^Office of the Director, Deputy Director for Non Infectious Diseases, CDC.

Marijuana is the most commonly used illicit substance under federal law in the United States (*1*); however, many states have legalized medical and adult nonmedical use. Evidence regarding the safety and health effects of cannabis use during pregnancy is largely inconclusive (*2*). Potential adverse health effects to exposed infants (e.g., lower birthweight) have been documented (*2*). To provide population-based estimates of use surrounding pregnancy, identify reasons for and mode of use, and understand characteristics of women who continue versus cease marijuana use during pregnancy, CDC analyzed data from eight states participating in the 2017 Pregnancy Risk Assessment Monitoring System (PRAMS) marijuana supplement. Overall, 9.8% of women self-reported marijuana use before pregnancy, 4.2% during pregnancy, and 5.5% after pregnancy. The most common reasons for use during pregnancy were to relieve stress or anxiety, nausea or vomiting, and pain. Smoking was the most common mode of use. In multivariable models that included age, race/ethnicity, marital status, education, insurance status, parity, trimester of entry into prenatal care, and cigarette and e-cigarette use during pregnancy, women who continued versus ceased marijuana use during pregnancy were more likely to be non-Hispanic white or other race/ethnicity than non-Hispanic black, be unmarried, have ≤12 years of education, and use cigarettes during pregnancy. The American College of Obstetricians and Gynecologists (ACOG) and the American Academy of Pediatrics (AAP) recommend refraining from marijuana use during pregnancy and lactation (*3*,*4*). Given the increasing number of states legalizing medical and adult nonmedical marijuana use, surveillance of perinatal marijuana use can inform clinical guidance, provider and patient education, and public health programs to support evidence-based approaches to addressing substance use.

PRAMS is a state-specific, population-based surveillance system designed to monitor self-reported behaviors and experiences before, during, and after pregnancy among women who have had a recent live birth. In each participating state, a monthly stratified systematic sample of women with recent live births is selected from birth certificate records and surveyed by mail or telephone 2–6 months after delivery.* Supplementary questions about marijuana use were asked in eight states included in this analysis: Alaska, Illinois, Maine, New Mexico, New York, North Dakota, Pennsylvania, and West Virginia; each state had a response rate ≥55%. Data were weighted to adjust for noncoverage and nonresponse and represent the total population of women with a live birth in each state in 2017.

Women were asked “At any time during the 3 months before you got pregnant or during your most recent pregnancy, did you use marijuana or hash in any form?” Use before pregnancy was identified as a frequency greater than “never” to the follow-up question “During the 3 months before you got pregnant, about how often did you use marijuana products in an average month?” Use during pregnancy was identified the same way, from the question “During your most recent pregnancy, about how often did you use marijuana products in an average month?” Women who indicated marijuana use in both periods were defined as having continued use, whereas those who used before pregnancy and ceased during pregnancy were defined as having ceased use. Women who indicated “yes” to the question “Since your new baby was born, have you used marijuana or hash in any form?” were defined as using marijuana after pregnancy. Women who self-reported use during pregnancy indicated the reason or reasons (to relieve nausea or vomiting; stress or anxiety; symptoms of a chronic condition; pain; to have fun or relax; and other) and mode or modes (smoking; eating; drinking; vaporizing; dabbing; or other) of using marijuana during pregnancy. More than one option could be chosen. Qualitative thematic coding categorized “other” responses; written responses of mental health conditions were recoded as relieving stress or anxiety and written responses of poor appetite or weight loss were recoded as relieving nausea or vomiting. Remaining responses were retained as other. Weighted prevalence estimates and 95% confidence intervals (CIs) were calculated overall and by state using SUDAAN (version 11.0; RTI International). Among women who used marijuana in the 3 months before pregnancy, chi-squared tests were used to compare characteristics of women who continued versus ceased marijuana use during pregnancy, including age, race/ethnicity, marital status, education, insurance status, parity, trimester of entry into prenatal care, and cigarette and e-cigarette use during pregnancy. Adjusted prevalence ratios (aPRs) were calculated to describe associations between continued versus ceased use in pregnancy and maternal characteristics. P-values <0.05 were considered significant.

Among 7,688 women, 6,236 (81.1%) had any information on marijuana use before, during, or after pregnancy. Prevalences of self-reported marijuana use before, during, and after pregnancy were 9.8%, 4.2%, and 5.5%, respectively (Figure 1). Estimates also varied by state, ranging from 8.4% in New York to 21.2% in Maine before pregnancy, 2.6% in New York to 12.1% in Maine during pregnancy, and 4.4% in Illinois to 15.9% in Maine after pregnancy. Among 413 women who reported use during pregnancy and their reason for use, the most commonly reported reasons included to relieve stress or anxiety (81.5%), nausea or vomiting (77.8%), and pain (55.1%) (Figure 2). Additional reported reasons included to have fun or relax (45.7%), relieve symptoms of a chronic condition (24.9%), and other (5.1%). The most common mode of marijuana use during pregnancy was smoking (91.0%); less frequently reported were eating (12.1%), vaporizing (7.1%), dabbing (4.5%), drinking (0.5%), and other (0.5%) modes. Among 765 women for whom data were available on marijuana use before and during pregnancy, 41.2% continued use, and 58.8% ceased use during pregnancy (Table). In multivariable analysis, women who continued versus ceased use during pregnancy were more likely to be non-Hispanic white (aPR = 1.8; 95% CI = 1.1–3.2) or other race/ethnicity (aPR = 2.5; 95% CI = 1.4–4.5) compared with non-Hispanic black, to be unmarried (aPR = 1.7; 95% CI = 1.1–2.6), have <12 years of education (aPR = 1.9; 95% CI = 1.3–2.8) or 12 years of education (aPR = 1.6; 95% CI = 1.1–2.2), compared with >12 years of education, and to have used cigarettes during pregnancy (aPR = 1.6; 95% CI = 1.2–2.3).

**FIGURE 1:**
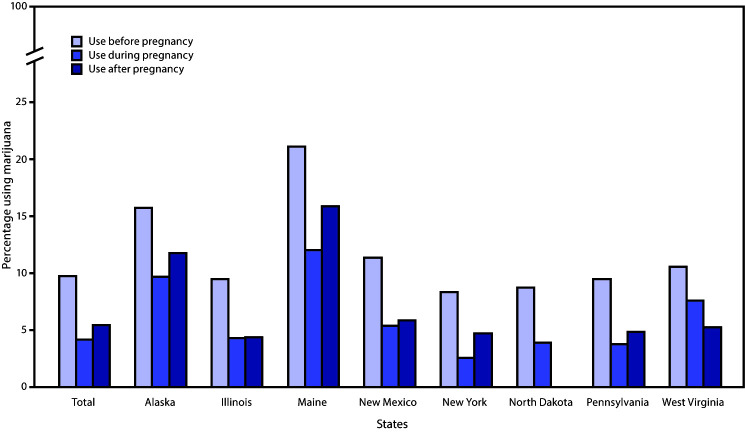
Prevalence* of marijuana use before, during, and after pregnancy (N = 6,236)**^†^** — eight states, Pregnancy Risk Assessment Monitoring System (PRAMS), 2017**^§^****^,^****^¶^** * Age-standardized prevalence estimates were also calculated but did not differ meaningfully from unadjusted results. ^†^ A total of 6,236 unique women had data on use before (n = 5,802), during (n = 5,805), and after pregnancy (n = 5,720). ^§^ Postpartum use estimates are not available for North Dakota. ^¶^ Marijuana legalization status as of 2017: medical and adult nonmedical use for Alaska and Maine; medical use for Illinois, New Mexico, New York, North Dakota, Pennsylvania, and West Virginia. In North Dakota and West Virginia, medical use of marijuana was legalized in 2017 but enactment might not have occurred by the time of PRAMS data collection.

**FIGURE 2:**
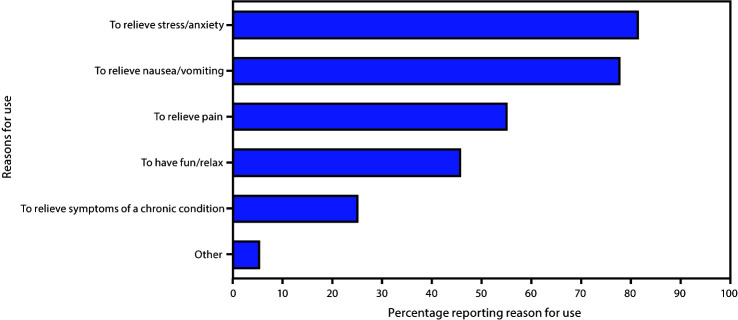
Reasons for marijuana use during pregnancy*^,†,§^ (N = 413) — eight states,**^¶^** Pregnancy Risk Assessment Monitoring System, 2017

* Among 418 women who used marijuana during pregnancy, five did not provide a response to reasons for use. More than one reason for use ccould be chosen.

^†^ To relieve stress/anxiety also includes written-in responses of to relieve “depression,” “anxiety,” “posttraumatic stress disorder,” “bipolar disorder,” and “conversion disorder.”

^§^ To relieve nausea/vomiting also includes written-in responses of “to increase appetite,” “to be able to eat,” and “to gain weight.”

^¶^ Alaska, Illinois, Maine, New Mexico, New York, North Dakota, Pennsylvania, and West Virginia.

**TABLE:** Maternal characteristics by marijuana use and cessation during pregnancy among women who used marijuana before pregnancy – eight states,* Pregnancy Risk Assessment Monitoring System (PRAMS), 2017

Maternal characteristic	Prepregnancy use weighted % (95% CI) (n = 765)^†^	Marijuana use status during pregnancy weighted % (95% CI)		aPR^§^ (95% CI)
		Continuous use (n = 410)^†^	Cease use (n = 355)^†^	
**Total**	—	**41.2 (35.1–47.6)**	**58.8 (52.4–64.9)**	—
**Age group, yrs**				
<20	7.9 (4.8–12.6)	45.4 (23.5–69.2)	54.6 (30.8–76.5)	1.0 (0.5–2.1)
20–24	26.3 (21.1–32.2)	52.6 (40.7–64.3)	47.4 (35.7–59.3)	1.2 (0.7–2.1)
25–34	54.3 (47.8–60.6)	37.5 (29.7–45.9)	62.5 (54.1–70.3)	1.2 (0.7–1.9)
≥35	11.6 (7.9–16.6)	30.2 (14.3–53.0)	69.8 (47.0–85.7)	Referent
**Race/Ethnicity**				
White, non-Hispanic	64.5 (57.8–70.7)	41.9 (34.5–49.5)	58.2 (50.5–65.5)	1.8 (1.1–3.2)^¶^
Black, non-Hispanic	15.4 (11.0–21.2)	28.3 (16.3–44.4)	71.7 (55.6–83.8)	Referent
Hispanic	13.6 (9.4–19.4)	41.4 (24.1–61.1)	58.6 (38.9–75.9)	1.7 (1.0–3.0)
Other**	6.5 (4.1–10.2)	70.0 (47.6–85.7)	30.0 (14.3–52.4)	2.5 (1.4–4.5)^¶^
**Marital status**				
Married	33.5 (27.8–39.6)	22.2 (15.4–31.1)^††^	77.8 (68.9–84.7)^††^	Referent
Not married	66.6 (60.4–72.2)	50.7 (42.5–58.9)^††^	49.3 (41.1–57.5)^††^	1.7 (1.1–2.6)^¶^
**Education, yrs**				
<12	13.6 (9.6–19.1)	75.1 (59.3–86.2)^††^	24.9 (13.8–40.7)^††^	1.9 (1.3–2.8)^¶^
12	30.1 (24.7–36.1)	58.0 (46.9–68.4)^††^	42.0 (31.6–53.1)^††^	1.6 (1.1–2.2)^¶^
>12	56.3 (49.8–62.5)	23.8 (17.8–31.2)^††^	76.2 (68.9–82.3)^††^	Referent
**Insurance status during prenatal care^§§^**				
Medicaid	51.2 (44.7–57.6)	53.8 (44.7–62.6)^††^	46.2 (37.4–55.3)^††^	1.0 (0.8–1.4)
Private^¶¶^	44.1 (37.8–50.6)	24.1 (17.1–32.9)^††^	75.9 (67.1–82.9)^††^	Referent
Other***	4.4 (2.0–9.2)	60.1 (25.8–86.7)^††^	39.9 (13.3–74.2)^††^	1.0 (0.4–2.7)
None	0.3 (0.2–0.7)	—^†††^	—^†††^	1.2 (0.7–2.2)
**Parity**				
First birth	47.6 (41.2–54.1)	34.9 (27.1–43.7)	65.1 (56.3–72.9)	Referent
Second or later birth	52.4 (45.9–58.8)	47.0 (38.2–56.0)	53.0 (44.0–61.8)	1.1 (0.8–1.4)
**Entry into prenatal care**				
First trimester	80.2 (74.4–85.0)	38.9 (31.9–46.5)	61.1 (53.5–68.1)	Referent
Second trimester	15.7 (11.4–21.2)	48.5 (32.5–64.9)	51.5 (35.1–67.6)	1.0 (0.7–1.5)
Third trimester/None	4.2 (2.3–7.5)	68.4 (36.6–89.1)	31.6 (10.9–63.4)	1.4 (0.9–2.3)
**Cigarette use during pregnancy^§§§^**				
Yes	29.6 (24.1–35.8)	73.7 (62.5–82.5)^††^	26.3 (17.5–37.5)^††^	1.6 (1.2–2.3)^¶^
No	70.4 (64.2–75.9)	27.6 (21.6–34.4)^††^	72.4 (65.6–78.4)^††^	Referent
**Electronic nicotine cigarette use during pregnancy**				
Yes	3.4 (1.8–6.2)	78.0 (45.8–93.7)	22.1 (6.3–54.2)	1.2 (0.6–2.1)
No	96.6 (93.8–98.2)	40.1 (33.9–46.6)	59.9 (53.4–66.1)	Referent

**Abbreviations:** aPR = adjusted prevalence ratio; CI = confidence interval.

* Alaska, Illinois, Maine, New Mexico, New York, North Dakota, Pennsylvania, and West Virginia.

^†^ Unweighted.

^§^ Among prepregnancy marijuana users, the association between continued use during pregnancy and maternal characteristics controlling for all other characteristics (unweighted n = 675).

^¶^ Adjusted prevalence ratio statistically significant (p<0.05).

** Other race/ethnicity includes persons who were not Hispanic and whose race was Alaska native, American Indian, Asian, Chinese, Filipino, Hawaiian, Japanese, other nonwhite, or mixed race.

^††^ Chi–square tests indicated that characteristic was significantly different (p<0.05) across marijuana use during pregnancy.

^§§^ Observations with no prenatal care or missing information on insurance during prenatal care were imputed with value for insurance during delivery.

^¶¶^ Includes Civilian Health and Medical Program of the Uniformed Services (CHAMPUS) and TRICARE.

*** Includes Children’s Health Insurance Program and other state government programs.

^†††^ Suppressed because cell sizes <5.

^§§§^ Ascertained from PRAMS and birth certificate.

## Discussion

Among women in eight states who had a recent live birth, 9.8% reported using marijuana before pregnancy, 4.2% during pregnancy, and 5.5% after pregnancy. The observed prevalence during pregnancy is similar to 2018 estimates from a national population-based survey, which found that 4.7% of pregnant women used marijuana in the preceding 30 days.^†^ The most common mode of marijuana use among those in the PRAMS sample was smoking, which is similar to findings from women attending prenatal care at two California medical centers (*5*). The most common reasons for use during pregnancy were to relieve stress or anxiety, nausea or vomiting, and pain. In a qualitative study, women who used marijuana during pregnancy reported that it helped them with nausea and appetite changes or improved their mood (*6*). Marijuana was also described by those women as natural and safe compared with other substances, including prescribed medications (*6*). In a national sample, approximately 70% of pregnant women perceived slight or no risk of harm from using marijuana once or twice a week (*7*). Among pregnant women who continued to use marijuana during pregnancy, 26% perceived it as harmful, whereas 75% of women who ceased use during pregnancy perceived it as harmful (*8*). In this analysis, after controlling for other factors, women who continued versus ceased use during pregnancy were more likely to be non-Hispanic white or other race/ethnicity than non-Hispanic black, be unmarried, have ≤12 years of education, and use cigarettes during pregnancy. Co-use of tobacco has been documented among pregnant women using marijuana (*7*) and is more likely to occur among pregnant women who continue to use marijuana than among those who cease use during pregnancy (*8*). Further, among pregnant women who reported drinking alcohol in the preceding 30 days, tobacco and marijuana were also commonly used (*9*). ACOG, AAP, and the United States Preventive Service Task Force recommend universal verbal screening during pregnancy to identify substance use (including marijuana) and provide opportunity for treatment when indicated (*3*,*4*,*10*).

ACOG and AAP recommend discontinuation of marijuana use during pregnancy and lactation because of insufficient pregnancy- and lactation-specific safety data (*3*,*4*). These guidelines also recommend that marijuana used for medicinal purposes be discontinued during pregnancy in favor of alternative therapies with better pregnancy-specific safety data (*3*,*4*). Marijuana is not currently regulated in the same manner as pharmaceuticals. Thus, even in states with comprehensive medical laws, plant-derived cannabis products might not have accurate dosing or content labels. Given the limited evidence surrounding the treatment effectiveness of marijuana, including a full understanding of potential harms during pregnancy, physicians and patients should discuss evidence-based pharmacologic and nonpharmacologic treatments during pregnancy.

The findings in this report are subject to at least four limitations. First, although data are state-specific population-based estimates of women who had live births in the eight states included in this report, these findings are not generalizable to populations in other states. Second, marijuana use was self-reported and might be biased because of social desirability and reporting requirements for substance use during pregnancy,^§^ as well as state legalization status of marijuana.^¶^ At the time of data collection in 2017, medical and adult nonmedical marijuana use was legal in Alaska and Maine, and medical use was legal in Illinois, New Mexico, New York, North Dakota, Pennsylvania, and West Virginia.** Third, written-in responses for other reasons of use were recoded to predetermined response options but might not be reflective of respondents’ intent. Finally, dependence on marijuana as a reason for use was not captured in this data source; in a national survey, 18.1% of pregnant women who used marijuana in the past-year met criteria for abuse or dependence (*7*).

Given the increasing number of states legalizing medical and nonmedical use of marijuana, surveillance of marijuana use in the perinatal period can inform clinical guidance, provider and patient education, and public health programs. Further monitoring of frequency, mode, and reasons for marijuana use during pregnancy could help characterize its use and inform research on adverse outcomes and prevention. Robust data are needed to inform effective policy and public health initiatives in the context of state legalization status. Provider education on evidence-based approaches for substance use screening during pregnancy and subsequent patient-provider discussions regarding common reasons for marijuana use during pregnancy might improve clinical care. Continued public education on the available science regarding the benefits and harms of cannabis use overall and during pregnancy is important.

**Summary**

**What is already known about this topic?**

Marijuana is an illicit substance under federal law; however, many states have legalized medical and nonmedical adult use. The American College of Obstetricians and Gynecologists and the American Academy of Pediatrics recommend refraining from marijuana use during pregnancy and lactation because evidence on safety and health effects are inconclusive or insufficient.

**What is added by this report?**

Overall, 9.8% of women reported marijuana use before pregnancy, 4.2% during pregnancy, and 5.5% after pregnancy. The most frequently reported reasons for marijuana use during pregnancy were to relieve stress or anxiety, nausea or vomiting, and pain.

**What are the implications for public health practice?**

Continuous surveillance of marijuana use in the perinatal period can inform clinical guidance, provider and patient education, and public health programs to support evidence-based approaches to addressing substance use.

## Footnotes

* https://www.cdc.gov/prams/methodology.htm.

† Table 6.18B, Substance Abuse and Mental Health Services Administration’s Results from the 2017 National Survey On Drug Use and Health. https://www.samhsa.gov/data/sites/default/files/cbhsq-reports/NSDUHDetailedTabs2017/NSDUHDetailedTabs2017.pdf.

§ https://www.guttmacher.org/state-policy/explore/substance-use-during-pregnancy.

¶ https://www.ncsl.org/research/health/state-medical-marijuana-laws.aspx.

** In North Dakota and West Virginia, medical use of marijuana was legalized in 2017 but enactment might not have occurred by the time of PRAMS data collection.
